# Arabic Translation and Psychometric Evaluation of the Depression Literacy Questionnaire among Adolescents

**DOI:** 10.1155/2016/8045262

**Published:** 2016-05-15

**Authors:** Hussain Ahmed Darraj, Mohamed Salih Mahfouz, Rashad Mohamed Al Sanosi, Mohammed Badedi, Abdullah Sabai, Abdulrahman AL Refaei, Hussain Mutawm

**Affiliations:** ^1^Jazan Health Affairs, Ministry of Health, Jazan 45911, Saudi Arabia; ^2^Family and Community Medicine Department, Faculty of Medicine, Jazan University, P.O. Box 2531, Jazan 45142, Saudi Arabia; ^3^Substance Abuse Research Centre (SARC), Jazan University, Jazan 45142, Saudi Arabia; ^4^Public Health Administration, Jazan Health Affairs, Ministry of Health, Jazan 82723, Saudi Arabia; ^5^Jazan General Directorate of Education, Ministry of Education, Jazan 82614, Saudi Arabia; ^6^Departments of English, Faculty of Languages and Translation, King Khalid University, Abha 62529, Saudi Arabia

## Abstract

*Background*. Depression is a serious mental health disease. Globally, it is estimated that almost 350 million people suffer from depression. It is important to assess depression literacy including knowledge and beliefs about mental disorders among adolescents.* Objective*. This study was conducted to validate the Arabic version of the Depression Literacy Questionnaire (D-Lit) among adolescents.* Methods*. A cross-sectional study was conducted among a sample size of 120 adolescents. Statistical analysis included face validation, confirmatory factor analysis, and reliability testing. A test-retest was carried out within a two-week interval.* Results*. The mean score for depression literacy among participants was 8.6 (SD = 4.48), the median was 8, and the interquartile range was 7. Preliminary construct validation analysis confirmed that factor analysis was appropriate for the Arabic version of D-Lit. The total internal consistency was measured by Cronbach's alpha coefficient and split-half test and the results were 0.78 and 0.71, respectively. The test-retest reliability measured by Pearson's correlation was 0.92 and spearman rho was equal to 0.91.* Conclusions*. Face validity, construct validity, and reliability analysis were found satisfactory for the Arabic version of D-Lit. The Arabic D-Lit was found valid and reliable to be used in the future studies.

## 1. Background

Depression is a serious mental health disease and generally characterized by sadness, loss of interest in activities, and decreased energy [1]. Evidence suggests that the onset of major depression predominantly manifests during adolescence stage. Furthermore, it influences a child's psychological, social, and academic functioning. Owing to that, they will be vulnerable to the risk of substance abuse and suicidal behavior [2, 3].

Depressive disorders in developing countries represent a key determinant of health-related disability. It is considered as a major source of nonfatal disease [4, 5]. Globally, it is estimated that almost 350 million people suffer from depression [6]. In the United Kingdom, it is reported that 60% of adolescents experienced depressive symptoms [7]. In the Eastern Mediterranean Region, the prevalence of mental disorders among children and adolescents was 10%–36%, which is either similar to or significantly higher than the global estimates [8]. In the neighboring country of the United Arab Emirates, just over 22% of children aged 6 to 18 years were reported to have mental health problems [9]. In Saudi Arabia, a recent study showed that the prevalence of depression among boys who are secondary school students in Abha city was approximately 38.2% [10]. Another study was conducted in the same region and showed that the prevalence of depression was 13.9% among girl students [11].

It is important to assess mental health literacy including knowledge and beliefs about mental disorders among adolescents [12]. A high level of mental health literacy on depression leads to a better understanding and outcomes of depressive disorders. A recent definition of mental health literacy is “the capacity to understand how to enhance and maintain good mental health; understand mental disorders and their treatments; decrease stigma against those living with a mental disorder and enhance help-seeking efficacy” [13]. Lack of mental health literacy results in a delay in seeking appropriate treatment and creates difficulties communicating with health professionals [14]. Schools are a typical venue in which to embed mental health literacy because the school students are more familiar with educational activities [15, 16].

One of the useful international assessment tools for mental health literacy specific to depression is the Depression Literacy Questionnaire (D-Lit) [17]. To date, Depression Literacy Questionnaire (D-Lit) validation studies have been made in numerous languages [17, 18]. Up to our best knowledge the Depression Literacy Questionnaire (D-Lit) has not so far been validated in Arabic.

The aim of the study was to translate the English-language version of the “Questionnaire on the Depression Literacy Questionnaire (D-Lit)” into Arabic and to validate the Arabic language version among adolescents so that it could be used in Arabic-speaking populations. Face validity, internal consistency, test-retest reliability, and construct validity were assessed.

## 2. Materials and Methods

### 2.1. The Instrument

The Depression Literacy Questionnaire (D-Lit) consists of 22 items, assessing the respondents' knowledge about depression. For each statement, respondents selected what they believed to be the correct response from three possible choices (true, false, or I don't know). There is a mix of true and false items in each scale. For example, “loss of confidence and poor self-esteem may be a symptom of depression” (D-Lit; true) and “people with depression disorder often hear voices that are not there” (D-Lit; false). Respondents' answers were scored 1 point for each correct answer, and the total score was ranged from 0 to 22. The higher score indicates a higher literacy toward depression.

### 2.2. Study Design and Participants

This study employed a cross-sectional study design. The source population was adolescent students in Jazan city. Four schools, two intermediate and two secondary schools, for boys and girls were randomly selected. The number of study participants for each phase was determined according to the type of validation method. 30 students were recruited in phase one which was focused on face validity, while in phase two, for construct validity, the sample size was calculated depending on Gorsuch's [19]. For reliability testing, the required sample size was calculated based on Cronbach's alpha formula. Taking into consideration 10% dropout, the final required sample size was 120 participants. In test-retest phase (phase three), the sample size was based on intraclass correlation coefficient (ICC) [20]. The minimally acceptable ICC value (*r*1 = 0.7) versus an alternative ICC value reflecting the expectations (*r*1 = 0.8) was chosen. The power was 80% and the significance level was 5%. The required sample size was 40 participants [20].

### 2.3. Translation and Cross-Cultural Adaptation

The planned procedures for translating the D-Lit were based on the guidelines of translation and cross-cultural adaptation by Beaton et al. [21]. Translation and back translation were conducted in order to confirm accuracy and appropriateness of Depression Literacy Questionnaire (D-Lit) wording. The instrument was translated by two independent persons from English into Arabic at the same time. One of them was aware of the study's purpose and goals, and the other one was not. Both translators had discussed the differences between their translations to resolve any differences until they developed a consensus about the Arabic wording of each item. Two back translations into English were done by two independent persons. The back translation was conducted with no prior exposure to the English-language version of the questionnaire. Then, Expert Committee Review was conducted. Principal investigator, translators, Arabic language expert, social expert, and psychiatrist discussed any discrepancies found between the original D-Lit and items and the back-translated versions the questionnaire. The committee also assessed the suitability of the instrument to be used for adolescents which was found appropriate. To avoid any limitation of the applicability of this version of the scale, the final translation was in classic Arabic, which can be used in other Arab countries with different dialects. The final Arabic version of the questionnaire is shown in Appendix B.

### 2.4. Data Collection

Data was collected from selected schools with no missing data. In the phase one, face validation was conducted. The selected participants (*n* = 30) were asked to go through the Arabic version of the D-Lit. After reading through the instrument, students were asked if they fully understood the instrument and its meaning. Participants reported clear understanding of the translated instrument.

In the second phase, construct validation and reliability testing were conducted. The Arabic version of the D-Lit was distributed to the participants (*n* = 120). The researcher briefly explained the content and how to answer the instrument before asking the participants to complete the instrument. The participants were encouraged to ask if they had any problem with the instrument. The average time taken to complete the instrument was 15 minutes. The completed instruments were returned to the researcher. At the end of phase two, no participants had indicated problems with the Arabic version of the D-Lit.

In phase three, test-retest reliability was conducted after a two-week interval. In this phase, we were able to reach 65 participants who had been involved in phase two and the same Arabic version of the D-Lit was given to them. The aim was to test if the participants would provide the same answer as in the previous phase.

### 2.5. Statistical Analysis

The collected data was analyzed using SPSS version 19.0. Descriptive statistics was used to summarize the demographic information and to obtain the descriptive details of depression literacy among the participants. In order to assess the factor structure of the translated version of the D-List prior to factor analysis, the preliminary analysis which indicates the adequacy of the instrument for factor analysis was evaluated. The preliminary analysis is represented by the value of the Kaiser-Meyer-Olkin (KMO) Measure of Sampling Adequacy, Individual Measure of Sampling Adequacy (MSA), and Bartlett's test of sphericity. The KMO value is expected to exceed the acceptable limit of 0.50 [22]. Lastly, Bartlett's test of sphericity indicates the appropriateness of factor analysis for the translated instrument [23]; thus it is expected to be significant. The analysis then proceeded with an assessment of the factor structure, since confirmatory factor analysis was conducted using SPSS application.

Assessment of the factor structure includes factor loading where items that are highly loaded into each factor were examined and then compared to previous studies. To assess the reliability of the Arabic version of the D-Lit, the internal consistency and test-retest reliability of the translated instrument were measured. The internal consistency reliability of the instruments is represented by Cronbach's alpha coefficient (*α*). Subsequently, Pearson's correlation coefficient (*R*) was calculated to evaluate the test-retest reliability. The correlation coefficient was calculated for the total score of the translated instrument.

### 2.6. Ethical Considerations

The study proposal and instrument were approved by the faculty of Medicine Ethical Committee. Authorization was granted from the headmasters of the selected schools. During the distribution of the questionnaire, students were told that the information collected would be kept anonymous and that participation was completely voluntary. Informed consent was sought from the eligible participants following full disclosure regarding the study before data collection is done. Proxy consent for children was obtained from parents or the person responsible. The purpose of the study was explained and participants were assured that they may withdraw from the study at any time during the study.

## 3. Results

### 3.1. Study Participants Background

Table 1 shows the background characteristics of the study population. A total of 120 students from 4 intermediate and secondary schools in Jazan city were involved in the study. Almost 88.3% (106) were Saudi, and 50% (60) were males.

### 3.2. Arabic-Translated Version of the D-Lit Scores

In the current study, the mean score for depression literacy among the participants was 8.6 (SD = 4.48), the median equals 8, the interquartile range was 7, and the range was 16. The descriptive summary of the Arabic-translated version of the D-Lit among the participants is tabulated in Table 2. Based on the mean score, the participants were divided into two groups: participants who scored below the mean score and participants who scored above the mean score. The percentage of those who scored above the mean score (46.6%) is shown to be less than those who scored below the mean score (53.4%).

### 3.3. Reliability Testing

The overall estimate of the reliability of the Arabic-translated version of the D-Lit (Cronbach's alpha) was 0.78 and split-half was equal to 0.71. The item-to-total score correlations were between *r* = 0.20 and *r* = 0.55, with 15 items exceeding the 0.30 criterion [24] and 7 items more than 0.2. The test-retest reliability of the total score, measured by Pearson's correlation, was scored and is found to be perfect *R* = 0.92 and Spearman rho was 0.91.

### 3.4. Readability Testing

Gunning Fog index is 6.26 for the Arabic version of D-Lit which indicates the number of years of formal education that a person requires in order to easily understand the questions in the D-Lit scale on the first reading.

### 3.5. Factor Analysis

**Figure 1:**
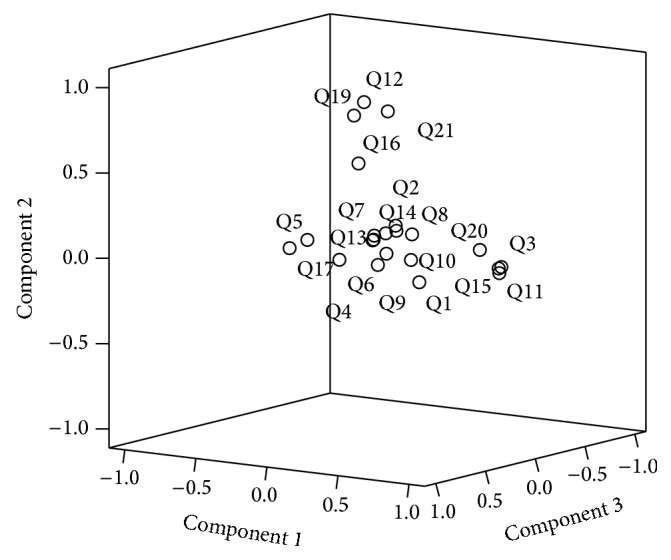
Component plot in rotated space.

The preliminary analysis for factor analysis of the Arabic-translated version of the D-Lit showed a satisfactory result. The determinant was >0.00001. The value of the KMO measure of sampling adequacy was 0.63, while Bartlett's test for sphericity was found highly significant (*p* < 0.001). The factor loading of the translated instrument is shown in Table 3. It confirms that all the items of the scale have been explained by a single factor (component 1). Multiple components have been emerged to indicate the possibility of other specific dimensions of this scale, but we are going to ignore it because our objective is to confirm the existing factor in the original scale. The screen plot also clearly suggested that the dimensions underlay the items of the scale (Figure 1).

As shown in Table 3, a component 1 extracted factor explained 100% of the variance in the items of the scale. Thus, factor rotation was not needed. All communality values were above 0.30, as recommended by Tabachnick and Fidell, 2001 [25].

## 4. Discussion

This study represents the first attempt to examine the reliability and the validity of an Arabic version of the 22-item Depression Literacy Questionnaire (D-Lit). The results provide solid support for the scale's reliability and validity among adolescent school students. Reliability was demonstrated through adequate estimates of internal consistency; Cronbach's alpha was estimated at 0.78, which exceeds the minimum criterion of 0.70 [26]. This internal consistency estimate is consistent with findings from other studies of D-Lit measures, which reported alpha of 0.7 in a sample of adults [17] and alphas of 0.88 and 0.92, respectively, in Greek and Italian versions of the D-Lit [18]. The test-retest reliability of the total score, measured by Pearson's correlation was found to be perfect (*R* = 0.92). Spearman rho was found to be 0.91, which was the same as the reliability for the English, Greek, and Italian versions of the D-Lit (alpha = 0.71, alpha = 0.80, and alpha = 0.78, resp.) [17, 18].

The corrected item-to-total correlations for 15 items exceeded the 0.30 criterion [24] and 7 items more than 0.2 suggesting the homogeneity of the measure and that each item was measuring a unique construct. This finding was not reported in the original English version of the D-Lit.

The findings of the factor analysis provide further support for the construct validity of the Arabic D-Lit. All items have been explained by a single factor which indicates that the Arabic version of the D-Lit is valid to measure depression literacy as one scale.

Gunning Fog index is 6.26 for the Arabic version of D-Lit which indicates the number of years of formal education a person requires to easily understand the questions in the D-Lit scale on the first reading. On the other hand, we have measured the Gunning Fog index for the original English version which was found to be 14.49. This further confirms that Arabic D-Lit is appropriate for the level of adolescent students.

## 5. Limitations

Around half *n* = 65 of participants dropped during the second phase of the study and hence did not fill the same questionnaire, compared to participants in the first time test *n* = 120; this dropout was due to midterm exams but still it was a statistically appropriate sample for test-retest as the minimal sample size was calculated to be 40 participants.

## 6. Conclusion

The results of the analysis of the psychometric properties of the Arabic version of the D-Lit in adolescent school students yield promising evidence that the 22-item D-Lit has acceptable reliability and validity. The findings also indicate that the D-Lit is potentially useful for assessing depression literacy that precedes the development and designing of depression literacy educational programs for adolescent school students and to monitor the effectiveness of these interventions to produce the desired change, which is important for combating depression stigma and improving help-seeking behavior.

## Figures and Tables

**Table 1:** Background characteristics of the students.

Characteristic	(*n*)	(%)
Nationality		
Saudi	106	88.3
Non-Saudi	14	11.7
Sex		
Male	60	50
Female	60	50
Grade		
Excellent	77	73.3
Very good	20	19
Good	7	6.7
Poor	1	1

Total	120	100

**Table 2:** Arabic-translated version of the D-Lit scores.

Mean score (SD)	8.6 (SD = 4.48)	
Measurements	Frequency (*n*)	Percentage (%)

Below mean score	64	53.4
Above mean score	56	46.6

**Table 3:** Results of factor analysis.

*N*	Item to total score correlation	Factor loading	Communalities
Q1	0.220	0.354	0.540
Q2	0.399	0.442	0.826
Q3	0.550	0.772	0.906
Q4	0.237	0.287	0.558
Q5	0.455	0.534	0.904
Q6	0.234	0.273	0.724
Q7	0.283	0.275	0.810
Q8	0.315	0.383	0.693
Q9	0.313	0.438	0.547
Q10	0.234	0.286	0.696
Q11	0.474	0.704	0.846
Q12	0.381	0.401	0.829
Q13	0.253	0.277	0.298
Q14	0.206	0.254	0.841
Q15	0.421	0.671	0.831
Q16	0.212	0.224	0.636
Q17	0.314	0.393	0.897
Q18	0.212	0.240	0.784
Q19	0.377	0.389	0.715
Q20	0.336	0.522	0.499
Q21	0.388	0.440	0.771
Q22	0.365	0.419	0.717

**Table 4:** D-Lit.

3	2	1	:‏ لا أعلم) لكل من الأسئلة التالية‏،‎خطأ‎ ،‎الرجاء اختيار الاجابة المناسبة (صح	*N*
لا أعلم	خطأ	صح	.الأشخاص الذين يعانون من الاكتئاب غالبا ما يتكلمون بطريقة مشوشة وغير مترابطة	1
لا أعلم	خطأ	صح	.خطأ‎ المصابون بالاكتئاب قد يشعرون بالذنب عندما لا يكونوا على	2
لا أعلم	خطأ	صح	.السلوك المتهور والطائش هو عرض من أعراض الاكتئاب‎	3
لا أعلم	خطأ	صح	.فقدان الثقة وضعف الثقة بالنفس قد يكونا من أعراض الاكتئاب‎	4
لا أعلم	خطأ	صح	.عدم المشي على التصدعات في الرصيف قد تكون علامة على الاكتئاب‎	5
لا أعلم	خطأ	صح	.كثيرا ما يسمعون أصواتا غير موجودة_‎‏الأشخاص الذين يعانون من الاكتئاب	6
لا أعلم	خطأ	صح	.النوم الكثير أو القليل جدا قد يكون علامة على الاكتئاب	7
لا أعلم	خطأ	صح	.اﻹفراط في تناول الطعام أو فقدان الرغبة في الغذاء قد يكون علامة على الاكتئاب	8
لا أعلم	خطأ	صح	.الاكتئاب لا يؤثر على ذاكرتك وتركيزك	9
لا أعلم	خطأ	صح	.وجود شخصيات متعدده ومختلفة في اﻹنسان قد تكون علامة على الاكتئاب	10
لا أعلم	خطأ	صح	.الاشخاص قد يتحركون بـبطء أكثر أو يصبحون منفعلون نتيجة لاكتئابهم	11
لا أعلم	خطأ	صح	.المعالج النفسي السريري يمكن أن يصف أدوية مضادات الاكتئاب	12
لا أعلم	خطأ	صح	.الاكتئاب المتوسط يعطل حياة الشخص بقدر التصلب المتعدد أو الصمـم	13
لا أعلم	خطأ	صح	.معظم الناس الذين يعانون من الاكتئاب يحتاجون إلى التنويم في المستشفى	14
لا أعلم	خطأ	صح	.العديد من الناس المشهورين قد عانوا من الاكتئاب	15
لا أعلم	خطأ	صح	.العديد من طرق علاج الاكتئاب هي أكثر فعالية من أدوية مضادات الاكتئاب	16
لا أعلم	خطأ	صح	.المشورة هي فعّالة مثل العلاج السلوكي المعرفي لـلاكتئاب	17
لا أعلم	خطأ	صح	.العلاج السلوكي المعرفي هو فعّال كما مضادات الاكتئاب في حالات الاكتئاب الخفيفة الى المتوسطة	18
لا أعلم	خطأ	صح	.من المرجـح أن تكون الفيتامينات هي أكثر فائدة لعلاج الاكتئاب من بين جميع العلاجات البديلة ونمط الحياة	19
لا أعلم	خطأ	صح	.الناس الذين يعانون من الاكتئاب يجب عليهم التوقف عن تناول مضادات الاكتئاب بمجرد أن يشعروا بالتحسن	20
لا أعلم	خطأ	صح	.مضادات الاكتئاب تسبـب الادمان	21
لا أعلم	خطأ	صح	.‎ﴽ‎مفعول الأدوية المضادة لـلاكتئاب عادة ما يظهر فور	22

## Appendix

### A. Depression Literacy Questionnaire Items (D-Lit)

Answer each item with one of three options – true, false or don't know.

1. People with depression often speak in a rambling and disjointed way.
  —
2. People with depression may feel guilty when they are not at fault.
  —
3. Reckless and foolhardy behavior is a common sign of depression.
  —
4. Loss of confidence and poor self-esteem may be a symptom of depression.
  —
5. Not stepping on cracks in the footpath may be a sign of depression.
  —
6. People with depression often hear voices that are not there.
  —
7. Sleeping too much or too little may be a sign of depression.
  —
8. Eating too much or losing interes1t in food may be a sign of depression.
  —
9. Depression does not affect your memory and concentration.
  —
10. Having several distinct personalities may be a sign of depression.
  —
11. People may move more slowly or become agitated as a result of their depression.
  —
12. Clinical psychologists can prescribe antidepressants.
  —
13. Moderate depression disrupts a person's life as much as multiple sclerosis or deafness.
  —
14. Most people with depression need to be hospitalized.
  —
15. Many famous people have suffered from depression.
  —
16. Many treatments for depression are more effective than antidepressants.
  —
17. Counseling is as effective as cognitive behavioral therapy for depression.
  —
18. Cognitive behavioral therapy is as effective as antidepressants for mild to moderate depression.
  —
19. Of all the alternative and lifestyle treatments for depression, vitamins are likely to be the most helpful.
  —
20. People with depression should stop taking antidepressants as soon as they feel better.
  —
21. Antidepressants are addictive.
  —
22. Antidepressant medications usually work straight away.
  —

### B. Arabic Version of the D-Lit

See Table 4.
